# Inhibition of ABA-induced stomatal closure by fusicoccin is associated with cytosolic acidification-mediated hydrogen peroxide removal

**DOI:** 10.1186/1999-3110-55-33

**Published:** 2014-03-17

**Authors:** Ai-Xia Huang, Xiao-Ping She, Jin-Liang Zhao, Yun-Ying Zhang

**Affiliations:** College of Life Sciences, Shaanxi Normal University, 710062 Xi’an, China

**Keywords:** Abscisic acid, Cytosolic acidification, Fusicoccin, Guard cell, Hydrogen peroxide

## Abstract

**Background:**

Fusicoccin (FC), a fungal phytotoxin produced by *Fusicoccum amygdale*, causes the inhibition of ABA-induced stomatal closure. The mechanism of inhibition is remaining unclear. We analyzed the role of hydrogen peroxide (H_2_O_2_) and relationship between H_2_O_2_ removal and cytosolic pH changes during inhibition of ABA-induced stomatal closure by FC.

**Results:**

According to the results, ABA treatment induced H_2_O_2_ production and stomatal closure, but FC inhibited the effects of ABA on these two parameters. Treatment with catalase (CAT) and NADPH oxidase inhibitor diphenylene iodonium (DPI) mimicked the effect of FC. These data suggest that inhibition of ABA effect by FC is related to the decrease of H_2_O_2_ levels in guard cells. Furthermore, similar to CAT, FC not only suppressed stomatal closure and H_2_O_2_ levels in guard cells treated with exogenous H_2_O_2_, but also reopened the stomata which had been closed by ABA and reduced the level of H_2_O_2_ that had been produced by ABA, indicating that FC causes H_2_O_2_ removal in guard cells. The butyric acid treatment simulated the effects of FC on the stomatal aperture and H_2_O_2_ levels in guard cells treated with exogenous H_2_O_2_ and had been closed by ABA, and both FC and butyric acid reduced cytosolic pH in guard cells of stomata treated with H_2_O_2_ and had been closed by ABA, which demonstrate that cytosolic acidification mediates FC-induced H_2_O_2_ removal.

**Conclusion:**

These results suggest that FC causes cytosolic acidification in guard cells, then induces H_2_O_2_ removal and reduces H_2_O_2_ levels in guard cells, finally inhibits stomatal closure induced by ABA.

**Electronic supplementary material:**

The online version of this article (doi:10.1186/1999-3110-55-33) contains supplementary material, which is available to authorized users.

## Background

Abscisic acid (ABA) is a phytohormone that plays vital roles in the control of growth and development and is involved in the response to various environmental stresses. ABA has been demonstrated to affect leaf size, shoot growth, stomatal and lateral root development (Parent *et al*. 2009; Finkelstein *et al*. 2002; Arend *et al*. 2009; Lenoble *et al*. 2004; de Smet *et al*. 2003). Moreover, it’s generally known ABA as a stress signal in plants. Drought and high salinity resulted in strong increases of plant ABA levels, accompanied by a major change in gene expression and in adaptive physiological responses (Christmann *et al*. 2007; Rabbani *et al*. 2003; Zeller *et al*. 2009). ABA has been shown to induce stomatal closure and reduce the loss of transpirational water from plants under drought conditions (García-Mata and Lamattina 2001; Luan 2002). A large number of ABA signaling intermediates has been identified in guard cells, including cytosolic calcium, protein kinases, cADPR, G proteins and ion channels (Hamilton *et al*. 2000; Schroeder *et al*. 2001; Leckie *et al*. 1998; Wang *et al*. 2001). Moreover, phospholipid sphingosine-1-phosphate (SIP), phospholipase C and nitric oxide (NO) have also been suggested to involve in ABA signaling pathways in stomatal closing movement (Hetherington 2001; Ng *et al*. 2001; Bright *et al*. 2006; Zhang *et al*. 2007).

Fusicoccin (FC), a fungal phytotoxin produced by *Fusicoccum amydali*, stimulates several physiological and biochemical processes, such as cell elongation, breaking of seed dormancy, ethylene production, stomatal opening and solute transport (Marrè 1979; Malerba *et al*. 1995). Since several lines of evidence obtained both in vivo and in vitro proved that FC is a powerful activator of the plasma membrane (PM) H^+^-ATPase, most of the effects induced by the toxin in the plant tissues have been ascribed to the activation of this transport system (Marrè 1979; Beffagna *et al*. 1977; Palmgren 1998). It is now widely accepted that FC activates the H^+^-ATPase by binding to a regulatory protein belonging to the 14-3-3 family, whose association with a specific binding sequence located at the end of the C-terminal autoinhibitory domain of the PM H^+^-ATPase releases the autoinhibitory action (Beffagna and Lutzu 2007). FC binding promotes and stabilizes this association, releases the autoinhibitory action and thus induces the activation of H^+^-ATPase (. Olsson *et al*^1998^; Svennelid *et al*. 1999; Kinoshita and Shimazaki 2001).

Previous studies demonstrate that ABA induces H_2_O_2_ production and partially blocks both blue light- and FC-dependent activation of H^+^-ATPases by decreasing in phosphorylation of H^+^-ATPase via H_2_O_2_ (Schroeder *et al*. 2001; Zhang *et al*. 2004; Goh *et al*. 1996), and cytosolic alkalinization is an early step preceding the production of reactive oxygen species (ROS) in the ABA-triggered signal cascade in guard cells (Suhita *et al*. 2004; Gonugunta *et al*. 2008; Islam *et al*. 2010; Gehring *et al*. 1997). However, Irving *et al*. reported that acidification of guard cell cytosol by kinetin, IAA or FC preceded stomatal opening (Irving *et al*. 1992), H^+^-ATPase could be activated by FC powerfully (Marrè 1979; Beffagna *et al*. 1977), and a decrease of endogenous H_2_O_2_ levels were associated with auxins- and cytokinins-induced stomatal opening (Song *et al*. 2006). These data indicate an opposite action of FC and ABA on cytosolic pH, H^+^-ATPase activity and stomatal movement, and it remains unclear whether or not this effect is related to the changes of H_2_O_2_ levels. In the present work, we found that the inhibition of ABA-induced stomatal closure by FC involves a decrease in H_2_O_2_ levels in guard cells of *Vicia faba*, and the decrease of H_2_O_2_ levels is mediated by cytosolic acidification.

## Methods

### Chemicals

Molecular probes 2′,7′-dichlorodihydrofluorescein diacetate (H_2_DCF-DA) was obtained from Biotium (Hayward, CA). The fluorescence probes of 2′,7′-bis(2-carboxyethyl)-5n-carboxy fluorescein-acetoxy methyl ester (BCECF-AM), ABA, fusicoccin (FC), catalase (CAT, from bovine liver), diphenylene iodonium (DPI), DMSO, Pluronic F-127, MES and butyric acid were purchased from Sigma-Aldrich (St Louis, MO). Unless stated otherwise, the remaining chemicals were of the highest analytical grade available from various suppliers of Chinese companies.

### Plant materials

Broad bean (*Vicia faba* L.) was grown in a controlled-environment plant growth chamber with a humidity of 80%, a photon flux density of 300 μmol m^-2^ s^-1^PAR generated by cool white fluorescent tubes (Philips, New York, NY), and an ambient temperature 25 ± 2°C with a 14-h light and 10-h dark cycle. The epidermis was peeled carefully from the abaxial surface of the youngest, fully expanded leaves of 4-week-old seedlings, and cut into pieces about 5 mm width and 5 mm lengths.

### Stomatal bioassay

Stomatal apertures were monitored by the method of McAinsh *et al*. (McAinsh *et al*. 1996) with slight modifications. To study the effects of FC, CAT and DPI on stomatal closure caused by ABA, freshly prepared abaxial epidermal strips were incubated in CO_2_-free MES/KCl buffer (10 mM MES/KOH, 50 mM KCl, 100 μM CaCl_2_, pH 6.15) with FC, CAT or DPI for 3 h with ABA under light conditions (300 μmol m^-2^ s^-1^) at 25 ± 2°C. Final stomatal apertures were recorded with a light microscope and an eyepiece graticule previously calibrated with a stage micrometer. To study the effects of FC, CAT and butyric acid on stomatal closure caused by exogenous H_2_O_2_, epidermal strips were incubated in MES/KCl buffer with H_2_O_2_ alone, or containing FC, CAT and different concentrations of butyric acid for 3 h, and then the stomatal apertures were recorded. To study the effects of FC, CAT and butyric acid on stomata that had been closed by ABA, strips were incubated in MES/KCl buffer for 3 h with ABA and were then treated with fresh buffer alone, or containing FC, CAT and different concentrations of butyric acid for another 1 h, final stomatal apertures were recorded.

To avoid any potential rhythmic effects on stomatal aperture, experiments were always started at the same time of the day. In each treatment, we scored 30 randomly selected apertures per replicate and treatments were repeated three times. The data presented are the means of 90 measurements ± s.e.

### Dyes loading of H_2_DCF-DA and BCECF-AM

H_2_O_2_ content and cytosolic pH of guard cells were monitored with H_2_DCF-DA and BCECF-AM, respectively, as previously described (Irving *et al*. 1992; Allan and Fluhr 1997) with minor modifications.

The epidermal strips were treated as described for stomatal bioassay section, and were then loaded with 50 μM H_2_DCF-DA (10 min) or 20 μM BCECF-AM (10 min), in Tris–KCl loading buffer (Tris 10 mM and KCl 50 mM, pH 7.2) containing 0.05% Pluronic F-127 in the dark at 25 ± 2°C. In experiment involving time-course monitoring of H_2_O_2_ levels and cytosolic pH in guard cells, the epidermal strips were treated with ABA for 3 h and then with FC for another 10, 20, 30, 40, 50, or 60 min before loading with probes.

### Laser-scanning confocal microscopy

After excess dye was washed off with fresh Tris–KCl loading buffer in darkness, TCS SP5 laser-scanning confocal microscopy (Leica Lasertechnik Gmbh, Heidelberg, Germany) was used to measure cytosolic pH or H_2_O_2_ content in guard cells of *Vicia faba* (excitation 488 nm, emission 505–530 nm, power 15%, PMT 959, pinhole 0.000036, zoom ~4, normal scanning speed, frame 512 × 512 pixels). Images acquired from the confocal microscope were analysed with Leica image software and Photoshop. To enable the comparison of changes in signal intensity, confocal images were taken under identical conditions (in manual setup) for all samples, and in each treatment we measured three epidermal strips, and the treatment was repeated three times. The selected confocal images represented the same results from three replications.

### Statistical analysis

Statistical analyses were performed by using a one-way ANOVA followed by the least significant difference (l.s.d.) test.

## Results

### FC inhibits stomatal closure caused by ABA and reduces ABA-induced H_2_O_2_ levels in guard cells

**Figure 1:**
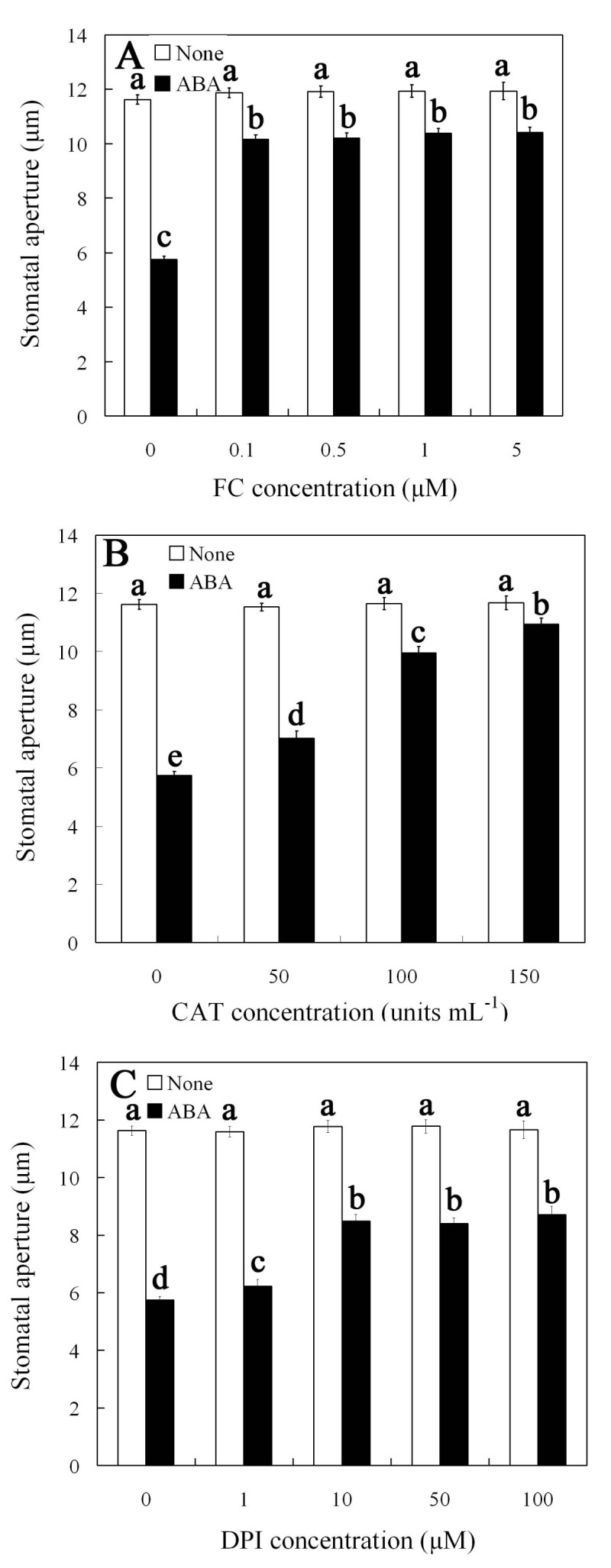
**FC inhibits ABA-induced stomatal closure.** Stomatal apertures were measured under light conditions (300 μmol m^-2^ s^-1^) at 25 ± 2°C. Values are the means of 90 measurements ± s.e. from three independent experiments. The asterisks in **(A)**, **(B)** and **(C)** indicate that the mean value is significantly different from that of the control at P <0.05 based on Fisher LSD post hoc test, respectively.

Previous studies have shown that FC, a fungal phytotoxin, causes irreversible stomatal opening (Assmann and Schwartz 1992; de Boer 1997). To gain insights into the effect of FC on ABA-induced stomatal closure, isolated epidermal strips of *V. faba* was incubated in different concentrations of FC with 10 μM ABA. As shown in Figure 1A, FC at concentrations of ≥ 0.1 μM obviously inhibited ABA-induced stomatal closure, so 0.1 μM FC was used in the following experiments.

Widely researches showed that ABA-induced stomatal closure is related to the production of endogenous H_2_O_2_ (Suhita *et al*. 2004; Pei *et al*. 2000; Murata *et al*. 2001). To know if there is a relationship between the inhibition of ABA-induced stomatal closure by FC and the levels of H_2_O_2_ in guard cells, the strips were treated with CAT (a H_2_O_2_ scavenger) and DPI (an inhibitor of H_2_O_2_–generating enzyme NADPH oxidase), respectively. The results show that CAT significantly inhibited ABA-induced stomatal closure in a dose-dependent manner (Figure 1B), and DPI suppressed ABA-induced stomatal closure partially (Figure 1C), indicating that H_2_O_2_ is required for ABA-induced stomatal closure and NADPH oxidase contributes to H_2_O_2_ production, which is consistent with the results reported previously (Zhang *et al*. 2001). The optimal concentration CAT and DPI on stomatal aperture were 100 units mL^-1^ and 10 μM, respectively. These results suggest that, probably like CAT and DPI, FC inhibition of ABA-induced stomatal closure via decreasing H_2_O_2_ levels in guard cells.

**Figure 2:**
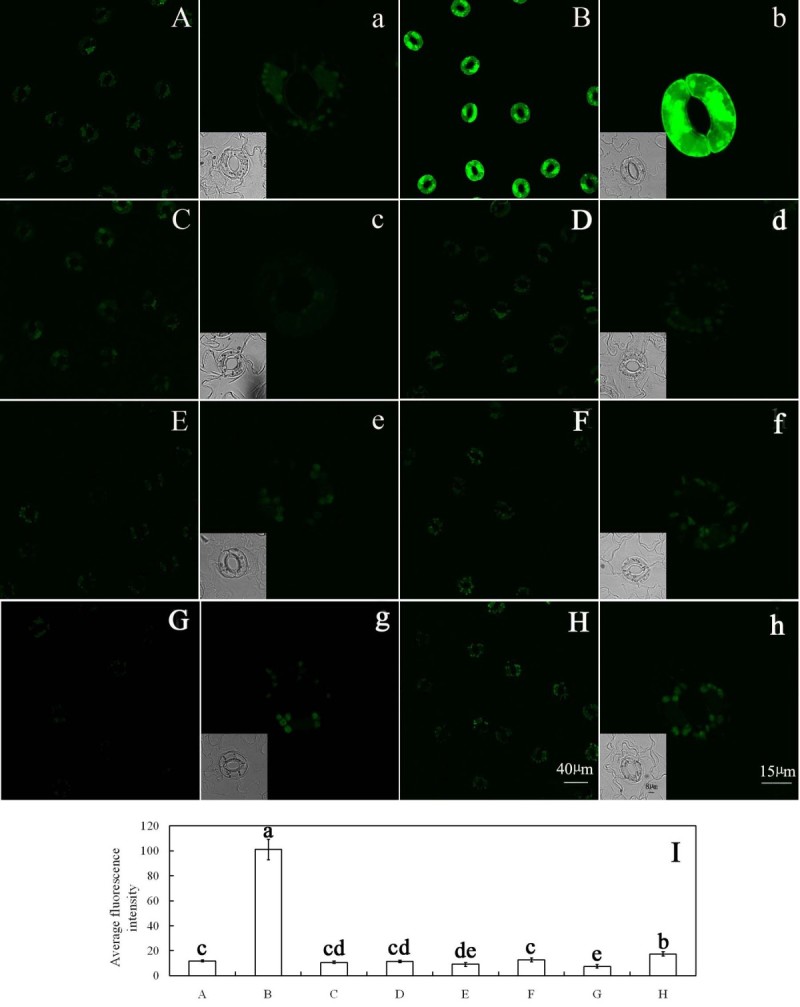
**FC reduces ABA-induced H**_**2**_**O**_**2**_**level in guard cells.** Guard cells of *Vicia faba* shown in image **(A)** were treated with CO_2_-free MES/KCl buffer alone for 3 h under light conditions (300 μmol m^-2^ s^-1^) at 25°C. Guard cells shown in image **(B)** were treated with 10 μM ABA, **(C)** with 0.1 μM FC, **(D)** 0.1 μM FC +10 μM ABA, **(E)** 100 units mL^-1^ CAT, **(F)** 100 units mL^-1^ CAT +10 μM ABA, **(G)** 10 μM DPI, **(H)** 10 μM DPI +10 μM ABA. **(I)** The average fluorescence intensity of guard cells in images **(A–H)**, data are means ± s.e. Values in **(I)** with different letters are significantly different at P <0.05 based on Fisher LSD post hoc test. The guard cells shown in image **(a–h)** are the representative of guard cells shown in image **(A–H)**. The insets show the bright-field images corresponding to the fluorescence images (a–h). Scale bars in image **(H)** and **(h)** represent 40 and 15 μm for images **(A–H)** and **(a–h)**, respectively. The bar in inset of image **(h)** represents 8 μm for all insets. Each experiment was repeated at least three times, and the selected confocal image represented the same results from approximately nine time measurements.

To further determine whether inhibition of ABA-induced stomatal closure by FC is accompanied by a decrease of H_2_O_2_ levels in guard cells, epidermal strips were loaded with H_2_DCF-DA, a specific probe for intracellular H_2_O_2_ (Allan and Fluhr 1997), to measure H_2_O_2_ levels directly in guard cells. As shown in Figure 2B, ABA induced an intense DCF fluorescence in guard cells, which is consistent with previous reports (Suhita *et al*. 2004; Pei *et al*. 2000; Murata *et al*. 2001). However, ABA-induced DCF fluorescence in guard cells was largely prevented by FC (Figure 2D). Similarly, treatment with CAT or DPI also substantially suppressed ABA-induced DCF fluorescence (Figure 2F,H). These results provide evidence that, like CAT and DPI, FC surely decreases H_2_O_2_ levels induced by ABA in guard cells.

### Both FC and butyric acid suppress exogenous H_2_O_2_-induced stomatal closure and DCF fluorescence in guard cells

**Figure 3:**
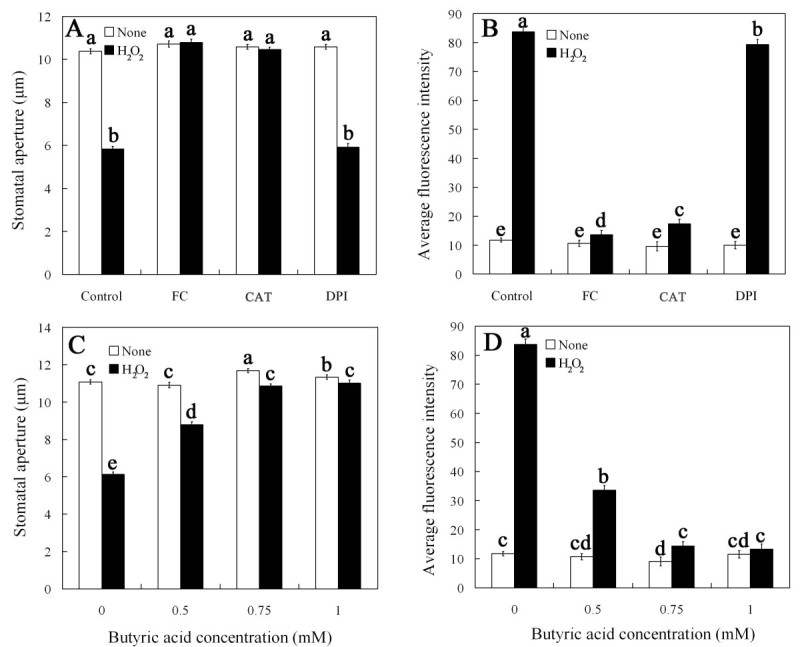
**FC and butyric acid suppress exogenous H**_**2**_**O**_**2**_**-induced stomatal closure (A, C) and reduces H**_**2**_**O**_**2**_**levels (B, D) in guard cells treated with exogenous H**_**2**_**O**_**2**_**.** Isolated epidermal strips were treated with 0.1 μM FC, 100 units mL^-1^ CAT, 10 μM DPI or different concentration of butyric acid (0, 0.5, 0.75, 1.0 mM) for 3 h with 100 μM H_2_O_2_ at 25°C. Other explanations are the same as in Figures 1 and 2.

Given that FC inhibition of ABA-induced stomatal closure is associated with a decrease of H_2_O_2_ levels in guard cells, we studied the pattern of H_2_O_2_ levels decreasing in response to FC. Epidermal strips were incubated in MES/KCl with H_2_O_2_ alone or containing FC, CAT or DPI for 3 h. As shown in Figure 3A, exogenous application of H_2_O_2_ obviously promoted stomatal closure, FC, CAT and DPI alone did not cause any changes of stomatal apertures. However, similar to CAT, FC significantly prevented stomatal closure induced by exogenous H_2_O_2_ (*P* < 0.05). DPI, an inhibitor of H_2_O_2_-generating enzyme NADPH oxidase, had no obvious effect on exogenous H_2_O_2_-induced stomatal closure (Figure 3A). The results indicate that FC decreases H_2_O_2_ levels probably via inducing H_2_O_2_ removal but not inhibiting the generation of H_2_O_2_, thereby preventing exogenous H_2_O_2_-induced stomatal closure.

To further clarify whether FC can affect exogenous H_2_O_2_-induced DCF fluorescence, the epidermal strips were treated with H_2_O_2_ in the presence of FC for 3 h, and then H_2_O_2_ levels were measured. As shown in Figure 3B, a striking DCF fluorescence in guard cells was observed after treatment with 100 μM H_2_O_2_. Compared with the control, there were no changes of DCF fluorescence in guard cells treated with FC alone (Figure 3B). However, H_2_O_2_-induced DCF fluorescence in guard cells was largely prevented by FC (Figure 3B). Similarly, CAT also substantially suppressed exogenous H_2_O_2_-induced DCF fluorescence (Figure 3B). DPI had no obvious effect on fluorescence (Figure 3B). These results show that like CAT, FC really induces H_2_O_2_ removal, and consequently reduces H_2_O_2_ level in guard cells treated with exogenous H_2_O_2_.

Previous study demonstrated that FC causes guard cells cytosolic acidification (Irving *et al*. 1992). These results prompt us to explore whether or not guard cells cytosolic acidification mediates H_2_O_2_ decrease induced by FC. For this purpose, we studied the effects of butyric acid on exogenous H_2_O_2_-induced stomatal closure and H_2_O_2_ levels in guard cells treated with exogenous H_2_O_2_. As shown in Figure 3C, butyric acid at the concentration of ≥ 0.5 mM significantly suppressed exogenous H_2_O_2_-induced stomatal closure (*P* < 0.05) (Figure 3C) and markedly reduced H_2_O_2_ contents in guard cells treated with exogenous H_2_O_2_ (*P* < 0.05) (Figure 3D). The results indicate that cytosolic acidification really promotes the removal of H_2_O_2_ within guard cells, thereby preventing H_2_O_2_-induced stomatal closure.

Both FC and butyric acid reopen the stomata had been closed by ABA and reduce the level of H_2_O_2_ had been generated by ABA in guard cells.

**Figure 4:**
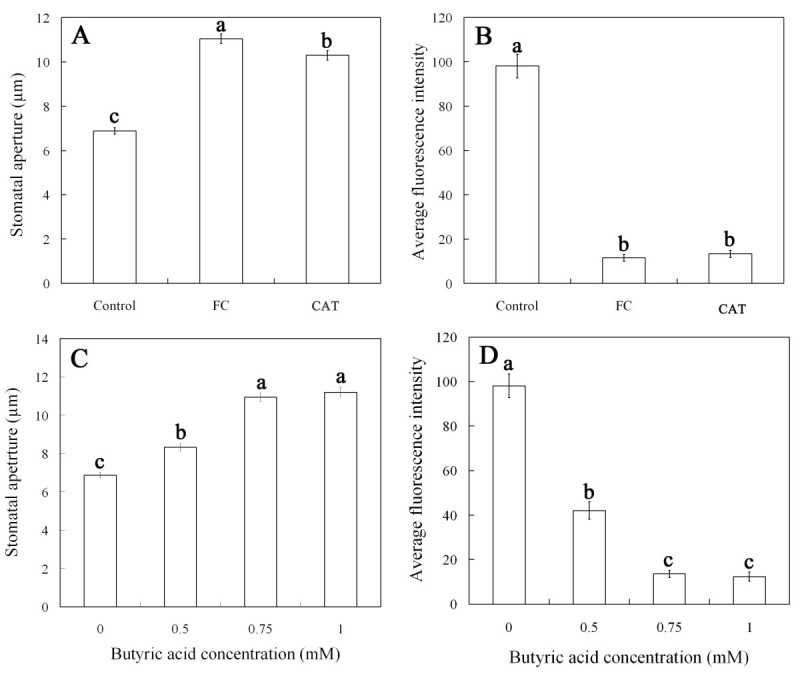
**FC and butyric acid reopens the stomata had been closed by ABA (A, C) and reduces the level of H**_**2**_**O**_**2**_**had been generated by ABA (B, D).** Strips of *Vicia faba* were incubated in CO_2_-free MES/KCl buffer for 3 h with 10 μM ABA and then treated with fresh MES/KCl buffer alone, or containing 0.1 μM FC, 100 units mL^-1^ CAT, 0.5, 0.75, 1 mM butyric acid, respectively, for another 1 h. Other explanations are the same as in Figures 1 and 2.

To further ascertain whether or not FC induces H_2_O_2_ removal, we also compared the effects of FC with CAT on the closed stomata induced by ABA and the level of H_2_O_2_ generated by ABA. As shown in Figure 4A, both FC and CAT obviously induced the closed stomata caused by ABA to reopen. The results indicate that, similar to CAT, FC induces the removal of H_2_O_2_ having been generated by ABA, thus resulting in stomatal reopening.

The effect of FC on the level of H_2_O_2_ that had been generated by ABA in guard cells was also measured in the present studies. After an incubation of 3 h in MES/KCl buffer in ABA, the strips were treated with fresh MES/KCl buffer alone, or containing FC and CAT for another 1 h, and then were loaded with H_2_DCF-DA, washed, and examined by laser scanning confocal microscopy. As shown in Figure 4B, compared with the control, both FC and CAT largely abolished the DCF fluorescence of guard cells. These results suggest that, the treatment of FC induces H_2_O_2_ removal, hence reduces the level of H_2_O_2_ induced by ABA in guard cells.

To further explore whether or not cytosolic acidification is related to the removal of H_2_O_2_ in guard cells, the effects of butyric acid on the stomata had been closed by ABA and the level of H_2_O_2_ had been generated by ABA were measured. Figure 4 shows that, butyric acid at the concentration of ≥ 0.5 mM evidently reopened the stomata had been closed by ABA (*P* < 0.05) (Figure 4C), and markedly reduced the level of H_2_O_2_ had been generated by ABA (*P* < 0.05) (Figure 4D). The results provide evidence that cytosolic acidification assuredly induces H_2_O_2_ removal in guard cells, and thus reopens the stomata had been closed by ABA.

Both FC and butyric acid reduce cytosolic pH in guard cells treated with exogenous H_2_O_2_ and stomata had been closed by ABA

**Figure 5:**
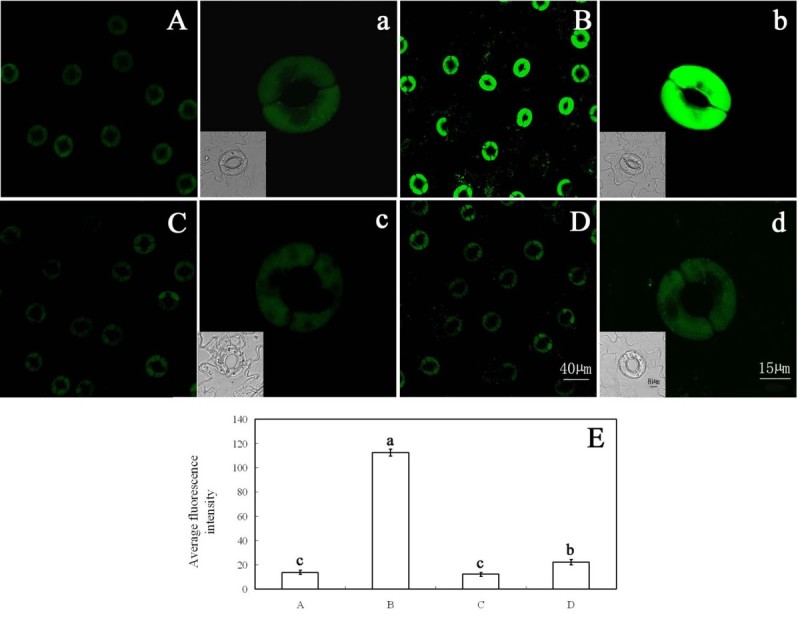
**FC reduces cytosolic pH in guard cells during ABA-induced stoamatal closure.** Guard cells of *Vicia faba* shown in image **(A)** were treated with MES/KCl buffer alone, **(B)** with 10 μM ABA, **(C)** 0.1 μM FC, **(D)** 0.1 μM FC +10 μM ABA for 3 h at 25°C. The guard cells shown in image **(a–d)** are the representative of guard cells shown in image **(A–D)**. **(E)** The average fluorescence intensity of guard cells in images **(A–D)**, data are means ± s.e. Values in **(D)** with different letters are significantly different at P <0.05 based on Fisher LSD post hoc test. Other explanations are the same as in Figure 2.

**Figure 6:**
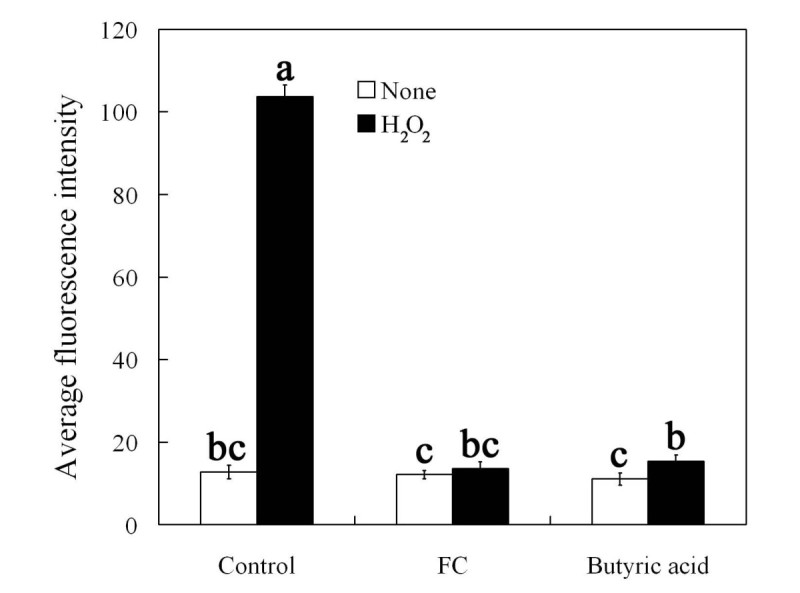
**FC and butyric acid reduces cytosol pH in guard cells treated with exogenous H**_**2**_**O**_**2**_**.** Isolated epidermal strips were incubated in MES/KCl buffer alone, or containing 0.1 μM FC, 0.75 mM butyric acid, 100 μM H_2_O_2_, 100 μM H_2_O_2_ + 0.1 μM FC, 100 μM H_2_O_2_ + 0.75 mM butyric acid for 3 h at 25°C. Other explanations are the same as in Figure 2.

**Figure 7:**
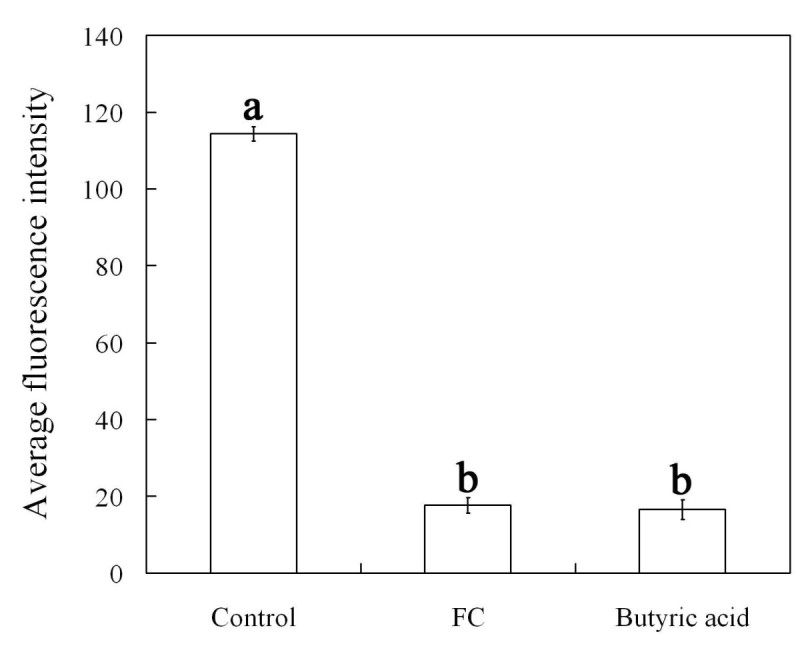
**FC and butyric acid reduces cytosolic pH in guard cells of stomata had been closed by ABA.** Strips of *Vicia faba* were incubated in MES/KCl buffer for 3 h with 10 μM ABA and then treated with fresh MES/KCl buffer alone, or containing 0.1 μM FC, 0.75 mM butyric acid for another 1 h. Other explanations are the same as in Figure 2.

To further determine whether or not cytosolic acidification in guard cells mediates FC-induced H_2_O_2_ removal, we measured the effect of FC on cytosolic pH in guard cells during ABA-induced stomatal closure, and the effects of FC and butyric acid on BCECF-AM fluorescence in guard cells treated with exogenous H_2_O_2_ and guard cells of stomata had been closed by ABA. As shown in Figure 5B, ABA obviously induced an increase of cytosolic pH in guard cells (*P* < 0.05), and ABA-induced increase of cytosolic pH was largely prevented by FC (*P* < 0.05) (Figure 5D). Figures 6 and 7 show that, 0.1 μM FC and 0.75 mM butyric acid significantly reduced cytosolic pH in guard cells treated with exogenous H_2_O_2_ and guard cells of stomata had been closed by ABA. The results confirm that FC surely causes cytosolic acidification, which is consistent with previous result (Irving *et al*. 1992). Together with the result that ABA- and exogenous H_2_O_2_- induced increase of H_2_O_2_ level was evidently reduced by FC and butyric acid (Figures 2D and 3B), the data from Figure 5, 6 and 7 suggest that FC-induced the decrease of H_2_O_2_ level is associated with guard cells cytosolic acidification during ABA-induced stomatal closure.

**Figure 8:**
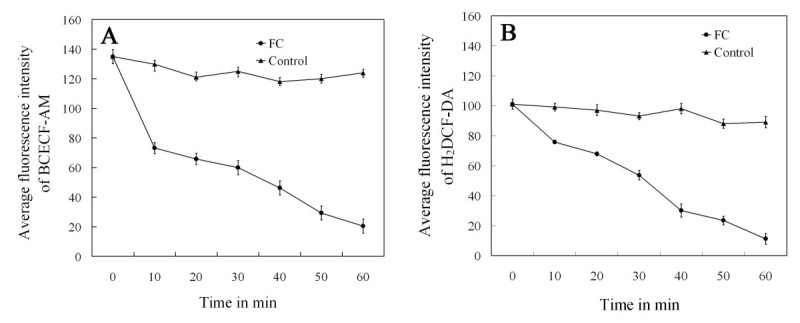
**Kinetics of decrease in cytosolic pH (A) or H**_**2**_**O**_**2**_**levels (B) in guard cells of stomata had been closed by ABA in response to 0.1 μM FC.** Epidermal strips were treated with 10 μM ABA for 3 h, and then incubated in 0.1 μM FC for 10–60 min. Other explanations are the same as in Figure 2.

Furthermore, we determined the kinetics of FC treatment on H_2_O_2_ levels or pH changes in guard cells of stomata had been closed by ABA. Treatment of ABA for 3 h caused a marked increase in both H_2_O_2_ levels and pH of guard cells (Figures 2B and 5B). When the strips were then treated with FC, the BCECF-AM fluorescence of guard cells decreased sharply after 10 min and declined to 54% (Figure 8A). Then BCECF-AM fluorescence dropped continually and reached minimum by 60 min (Figure 8A). In contrast, H_2_O_2_ levels of guard cells on exposure to FC were drop to 75% after 10 min (Figure 8B), then felled continually and reached minimum by 60 min (Figure 8B). Thus, the drop in pH of guard cells appeared to occur earlier to that of H_2_O_2_ levels decrease (Figure 8), confirming that cytosolic acidification precedes H_2_O_2_ removal during inhibition of ABA-induced stomatal closure by fusicoccin.

## Discussion

Guard cells control transpiration in plants and regulate gas exchange in leaves by opening and closing stomatal pores. Stomatal opening is induced by many abiotic and biotic factors, including light, indoleacetic acid (IAA), FC, cytokinins, low CO_2_ levels and high humidity (Mansfield and Atkinson 1990; Jewer and Incoll 1980; Pemadasa 1982; Braunsgaard *et al*. 1998), and stomatal closure can be promoted by dark, osmotic stress, high CO_2_ concentrations, decreased humidity and ABA (Schroeder *et al*. 2001; Kearns and Assmann 1993). Previous studies demonstrated that cytosolic alkalinization of guard cell precedes ROS production and is required for ABA- and MJ-induced stomatal closure (Suhita *et al*. 2004; Islam *et al*. 2010; Gehring *et al*. 1997; Gonugunta *et al*. 2009). FC, IAA and kinetin decreased the cytosolic pH and promoted stomatal opening (Irving *et al*. 1992), and a decrease of endogenous H_2_O_2_ levels were associated with auxins- and cytokinins-induced stomatal opening (Song *et al*. 2006). These results suggested that cytosolic pH was an important factor in the regulation of H_2_O_2_ levels and stomatal movement. However, until recently, little was known whether the inhibition of ABA-induced stomatal closure by FC is related to the change of cytosolic pH and H_2_O_2_ levels in guard cells. The results of the present studies demonstrate that similar to CAT and DPI, FC significantly prevented stomatal closure induced by ABA (Figure 1) and largely reduced H_2_O_2_ levels in guard cells induced by ABA (Figure 2). The results suggest that the inhibition of ABA-induced stomatal closure by FC is associated with a decrease of H_2_O_2_ levels in guard cells.

Previous reports demonstrated that FC stimulates production of H_2_O_2_ in cultured sycamore and *Arabidopsis thaliana* cells (Beffagna and Lutzu 2007; Malerba *et al*. 2003). However, FC was also reported to block cryptogein-induced H_2_O_2_ production of tobacco cells (Simon-Plas *et al*. 1997). The results of the present study show that the inhibition of ABA-induced stomatal closure by FC is associated with a decrease of H_2_O_2_ levels in stomatal guard cells. The question arises of how FC reduces H_2_O_2_ levels in guard cells. Our results show that FC suppressed exogenous H_2_O_2_-induced stomatal closure and H_2_O_2_ levels in guard cells treated with exogenous H_2_O_2_ (Figure 3A,B), and also reopened the closed stomata by ABA and abolished H_2_O_2_ that had been generated by ABA (Figure 4A,B). The above-mentioned effects of FC are similar to those of CAT (Figures 3A,B, and 4A,B), a scavenger of H_2_O_2_. These results prove that the treatment with FC induces the removal of H_2_O_2_ within guard cells, thereby prevents stomatal closure induced by exogenous H_2_O_2_, and causes the reopening of the closed stomata by ABA. Together with the facts that FC inhibits stomatal closure induced by ABA (Figure 1A) and reduces H_2_O_2_ levels in guard cells caused by ABA (Figure 2D), we conclude that FC probably initiates an unidentified mechanism, which can reduce H_2_O_2_ levels in guard cells via inducing H_2_O_2_ removal, eventually preventing stomatal closure induced by ABA.

Cytosolic pH is an important factor in the regulation of stomatal movement (Suhita *et al*. 2004; Gonugunta *et al*. 2008; Irving *et al*. 1992; Blatt 2000; Zhang *et al*. 2001). Cytosolic alkalinization is a major step in the ABA-triggered signal cascade in guard cells leading to stomatal closure (Irving *et al*. 1992; Blatt 2000), and further investigation found that cytosolic alkalinization preceded the production of ROS and NO during ABA induced stomatal closure (Suhita *et al*. 2004; Gonugunta *et al*. 2008, 2009). In contrast, FC, IAA or a weak acid butyrate, decreased the cytosolic pH and promoted stomatal opening (Irving *et al*. 1992), and auxins also reduce H_2_O_2_ levels (Song *et al*. 2006). These results prompted us to investigate the interaction of pH and H_2_O_2_ levels in FC-inhibited stomatal closure. In this study, we provide evidence that, similar to butyric acid, FC not only reduced cytosolic pH caused by ABA in guard cells (Figures 5 and 7) but also induced H_2_O_2_ removal (Figures 2 and 4), which suggests that cytosolic acidification in guard cells mediates H_2_O_2_ removal induced by FC. Real-time monitoring with the help of fluorescent dyes BCECF-AM and H_2_DCF-DA revealed that FC-induced decrease of cytosolic pH faster than that of H_2_O_2_ levels and confirmed that acidification of guard cell could be upstream of H_2_O_2_ during inhibition of ABA-induced stomatal closure by FC (Figure 8). Combined with the fact that FC reduces cytosolic pH (Figure 5D) and H_2_O_2_ levels (Figure 2D) in guard cells induced by ABA, we conclude that FC induces H_2_O_2_ removal via reducing cytosol pH, hence lessens H_2_O_2_ levels in guard cells during ABA-induced stomatal closure. It is an intriguing problem about how FC-induced cytosolic acidification causes H_2_O_2_ removal, and we suppose that some H_2_O_2_-scavenging mechanism might be activated, including change of ASA redox state, catalase or ascorbate peroxidase activities (Beffagna and Lutzu 2007; Chen and Gallie 2004).

## Conclusions

In summary, these data suggest that FC induces H_2_O_2_ removal and reduces H_2_O_2_ level via reducing cytosol pH in guard cells, thus inhibiting ABA-induced stomatal closure.

## Authors’ original submitted files for images

Below are the links to the authors’ original submitted files for images. Authors’ original file for figure 1 Authors’ original file for figure 2 Authors’ original file for figure 3 Authors’ original file for figure 4 Authors’ original file for figure 5 Authors’ original file for figure 6 Authors’ original file for figure 7 Authors’ original file for figure 8

## Abbreviations

ABA: Abscisic acid
CAT: Catalase
BCECF-AM: 2′,7′-bis(2-carboxyethyl)-5(6)-carboxy fluorescein-acetoxy methyl ester
DMSO: Dimethyl sulfoxide
DPI: Diphenylene iodonium
FC: Fusicoccin
H2DCF-DA: 2′,7′-dichlorodihydrofluorescein diacetate
H2O2: Hydrogen peroxide
MES: 2-(*N*-morpholino)ethanesulfonic acid.
