# Supplementary figures and images for: Inhibition of ABA-induced stomatal closure by fusicoccin is associated with cytosolic acidification-mediated hydrogen peroxide removal
Source: Bot Stud. 2014 Mar 17;55:33. doi: 10.1186/1999-3110-55-33 (PMC5432956; doi:10.1186/1999-3110-55-33)

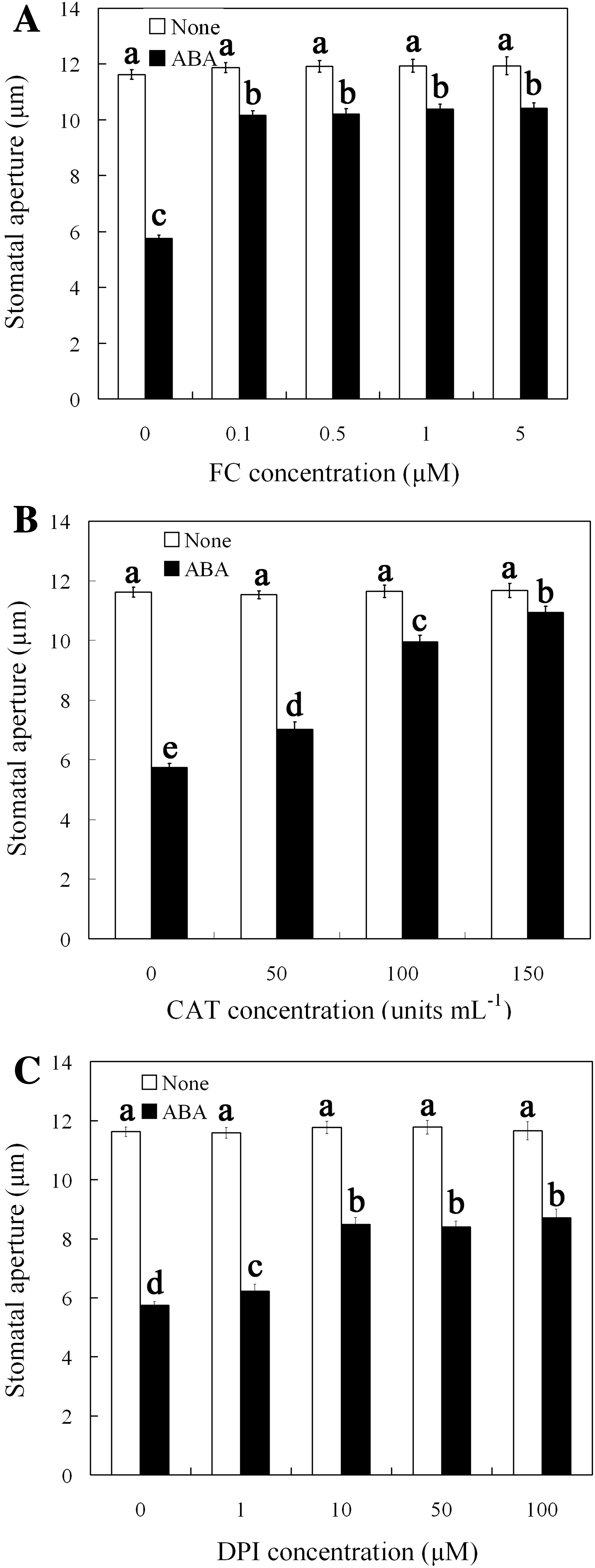

Supplement: Supplementary file 1 — Authors’ original file for figure 1 [file 40529_2012_89_MOESM1_ESM.tif]

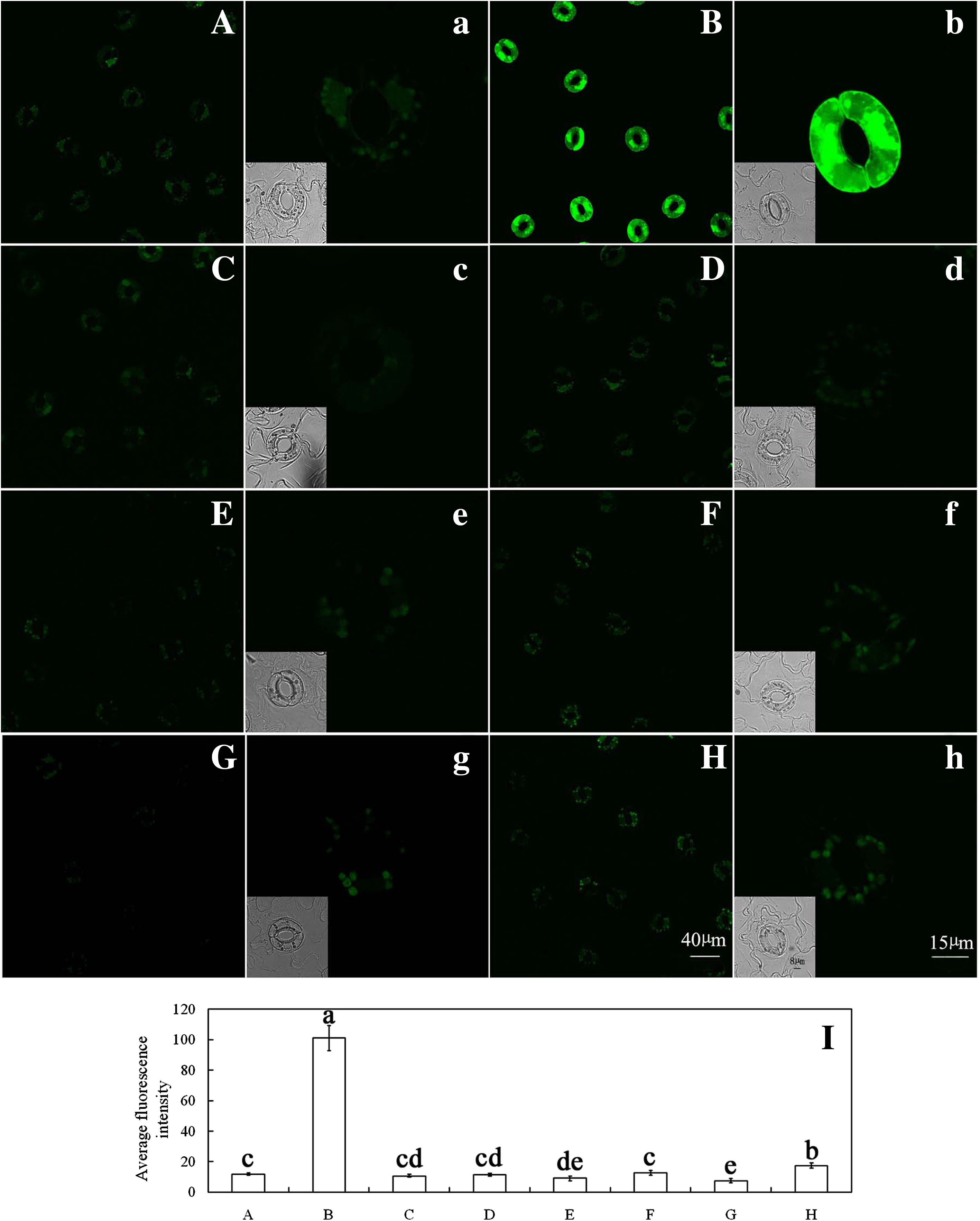

Supplement: Supplementary file 2 — Authors’ original file for figure 2 [file 40529_2012_89_MOESM2_ESM.tif]

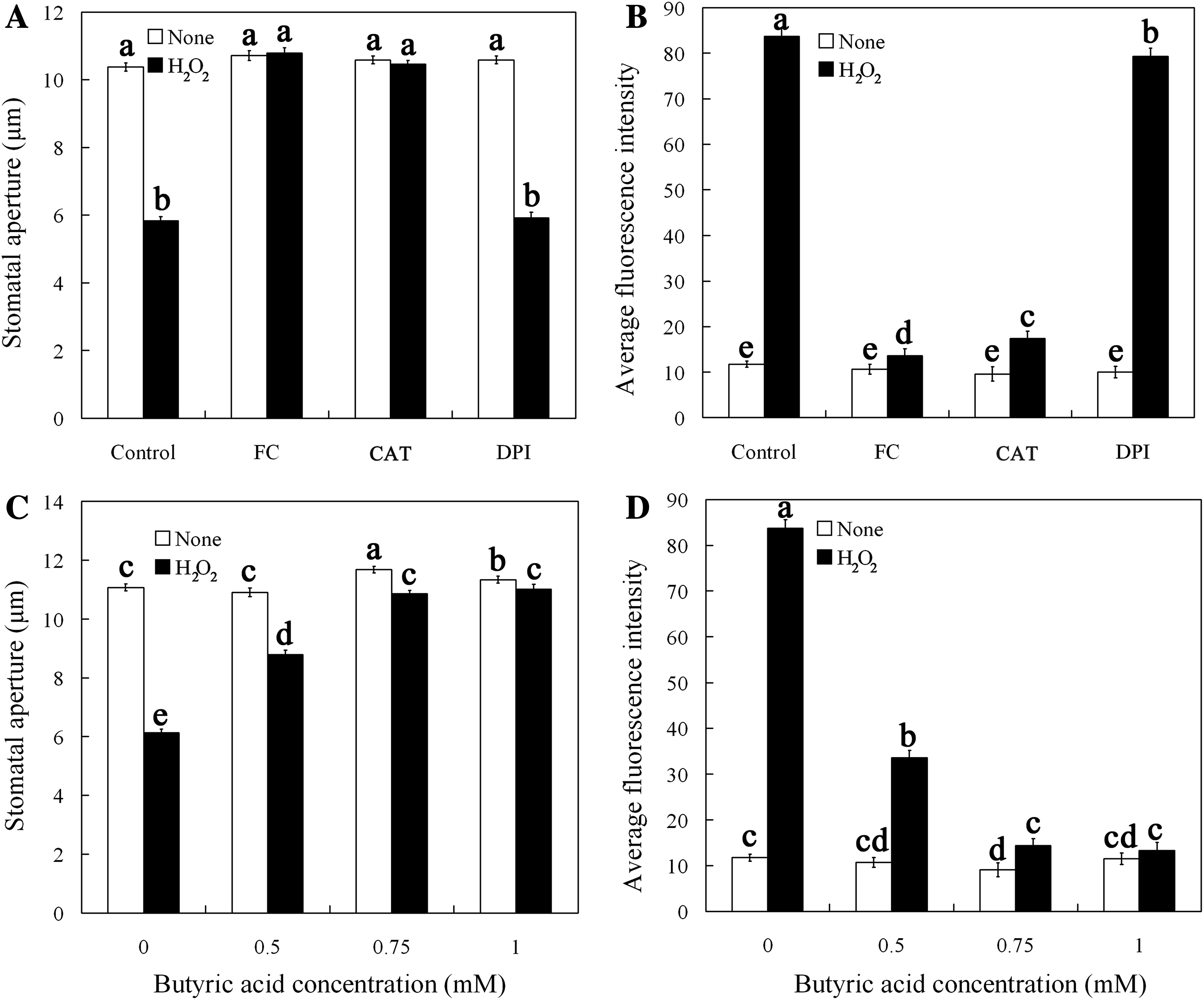

Supplement: Supplementary file 3 — Authors’ original file for figure 3 [file 40529_2012_89_MOESM3_ESM.tif]

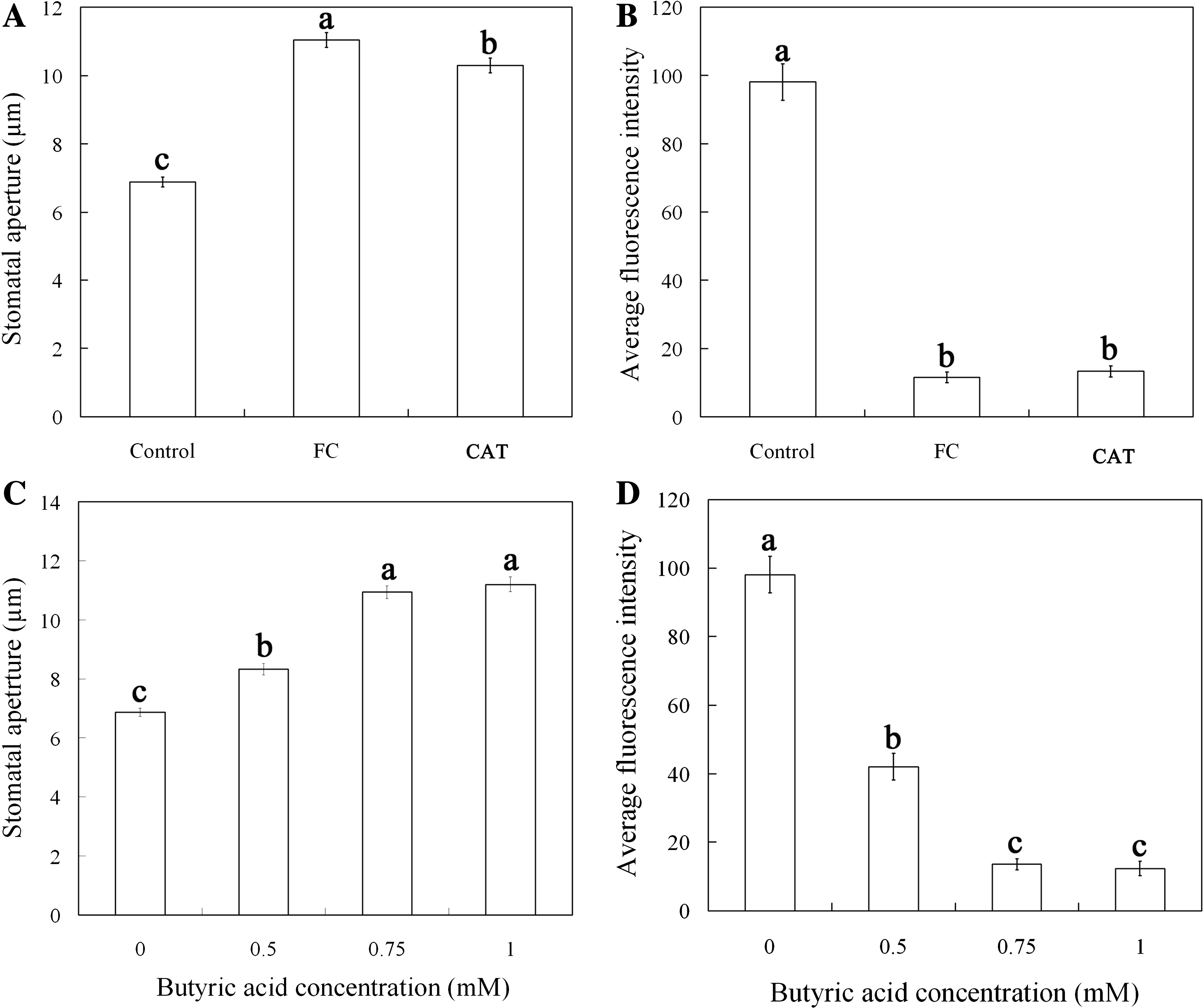

Supplement: Supplementary file 4 — Authors’ original file for figure 4 [file 40529_2012_89_MOESM4_ESM.tif]

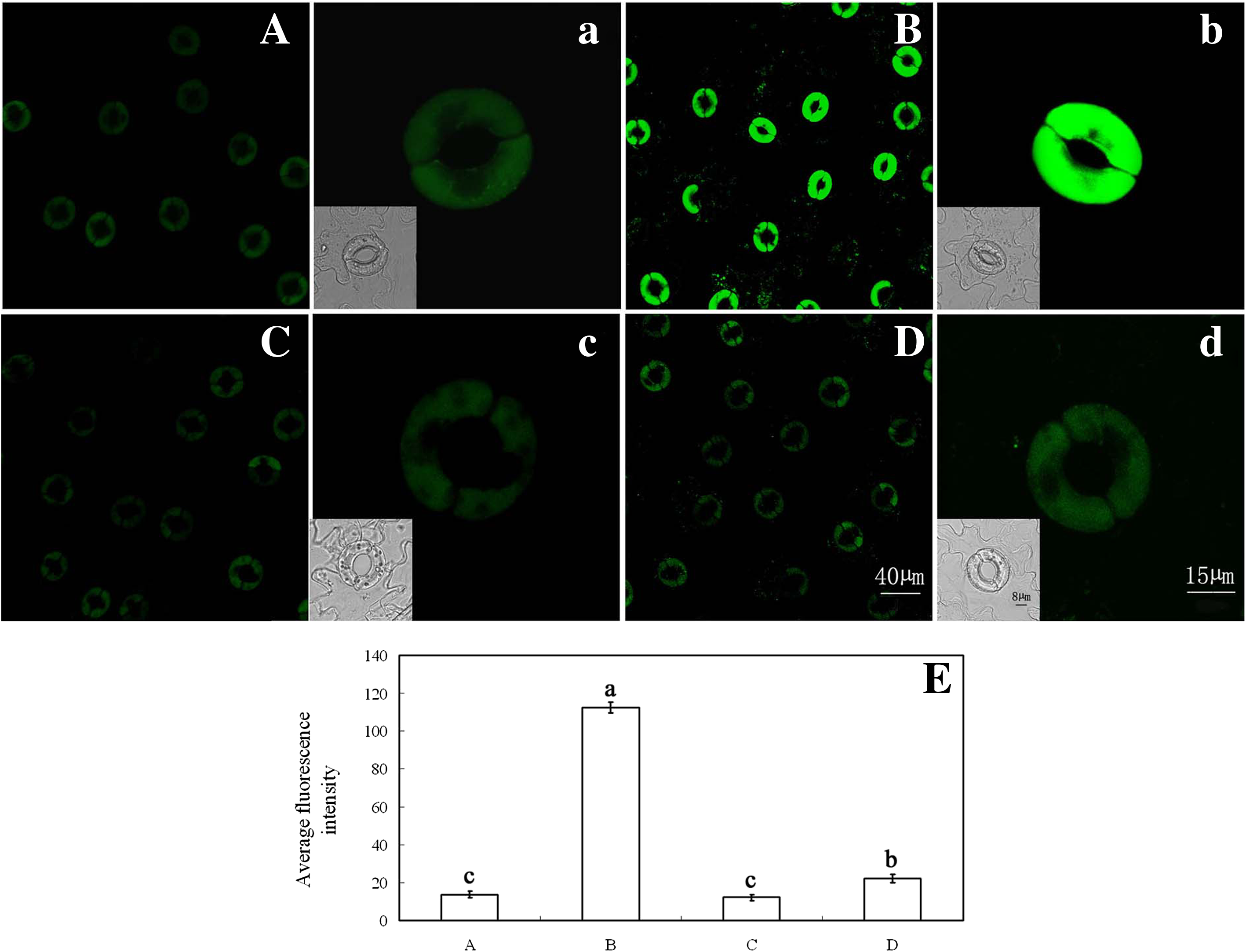

Supplement: Supplementary file 5 — Authors’ original file for figure 5 [file 40529_2012_89_MOESM5_ESM.tif]

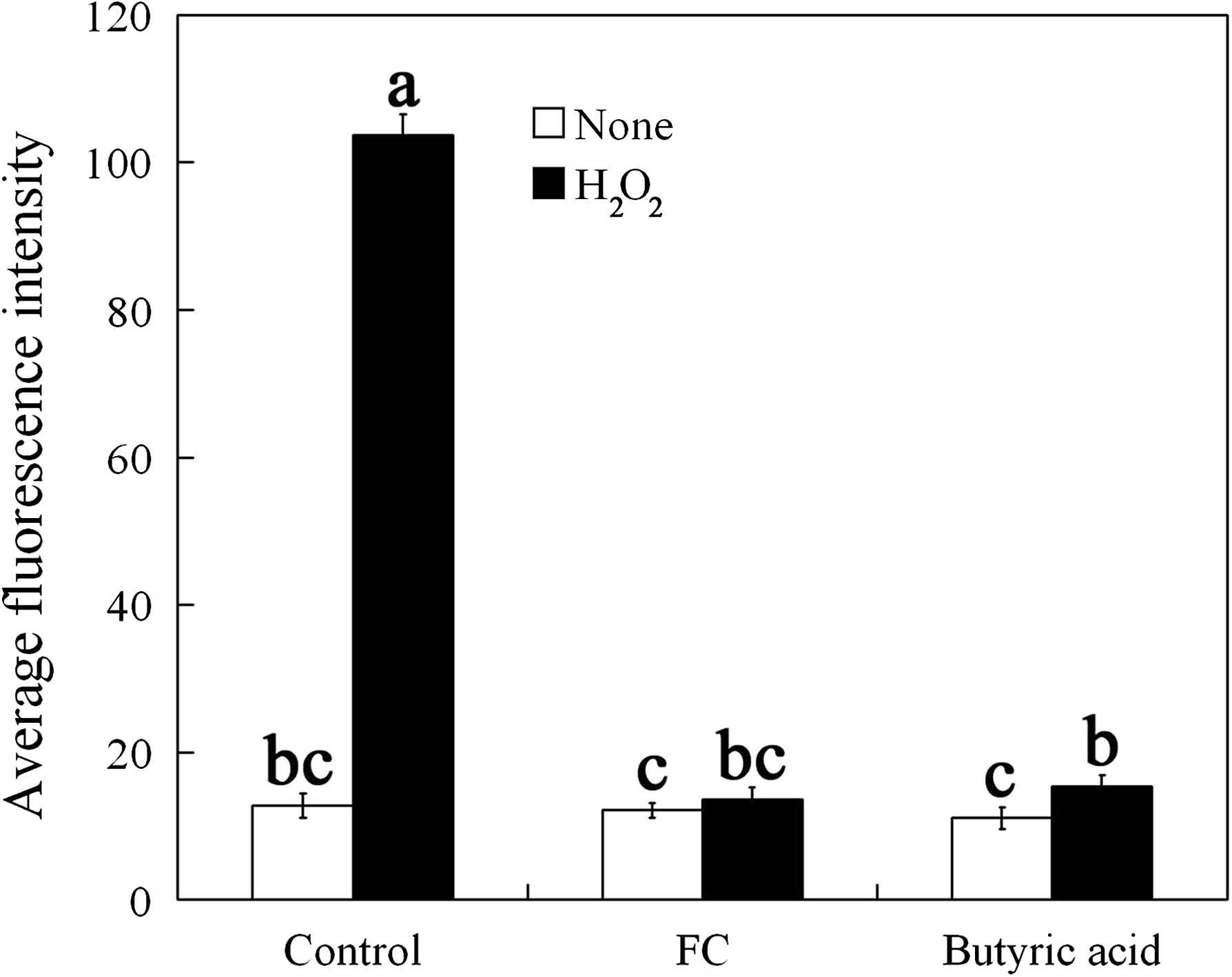

Supplement: Supplementary file 6 — Authors’ original file for figure 6 [file 40529_2012_89_MOESM6_ESM.tif]

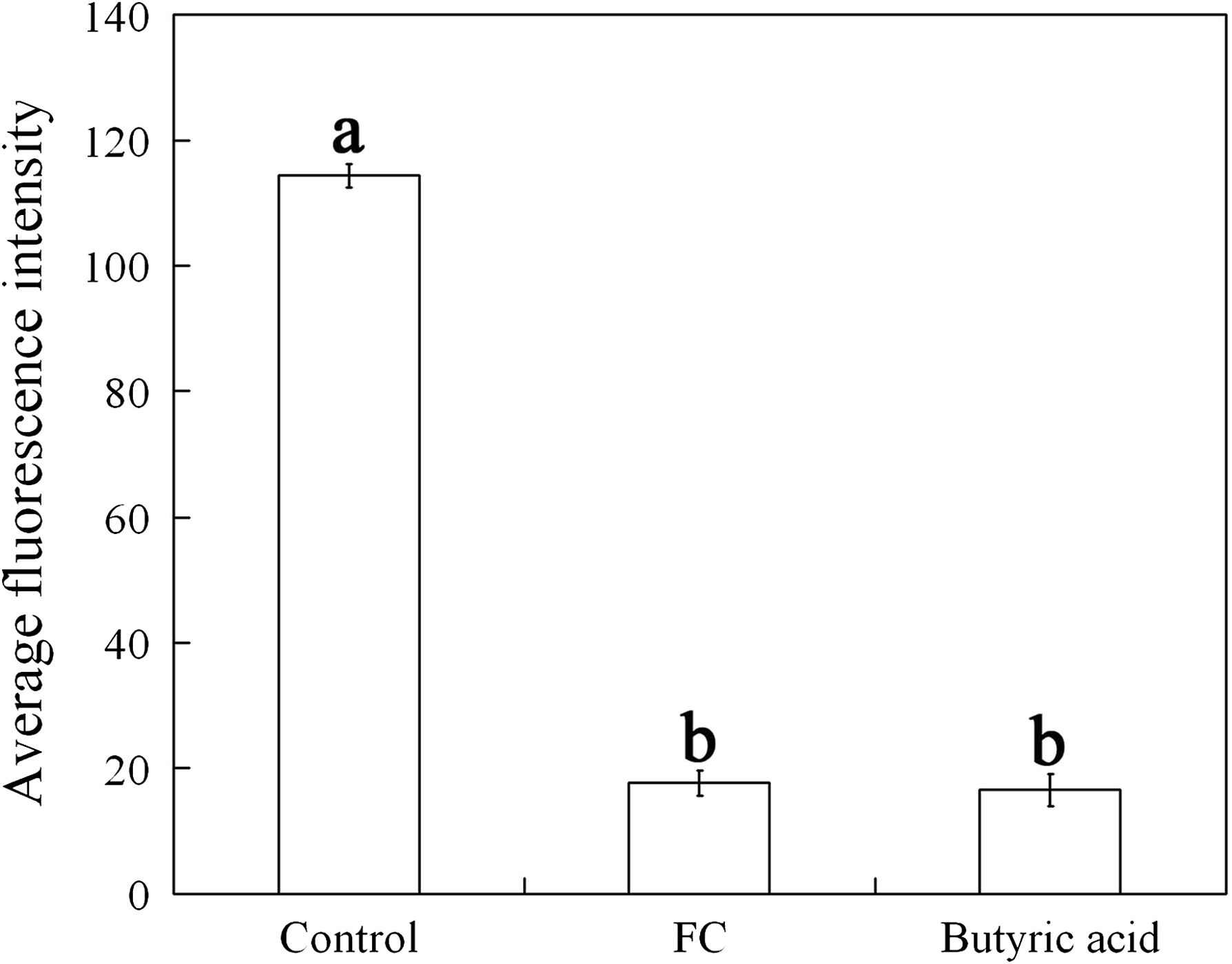

Supplement: Supplementary file 7 — Authors’ original file for figure 7 [file 40529_2012_89_MOESM7_ESM.tif]

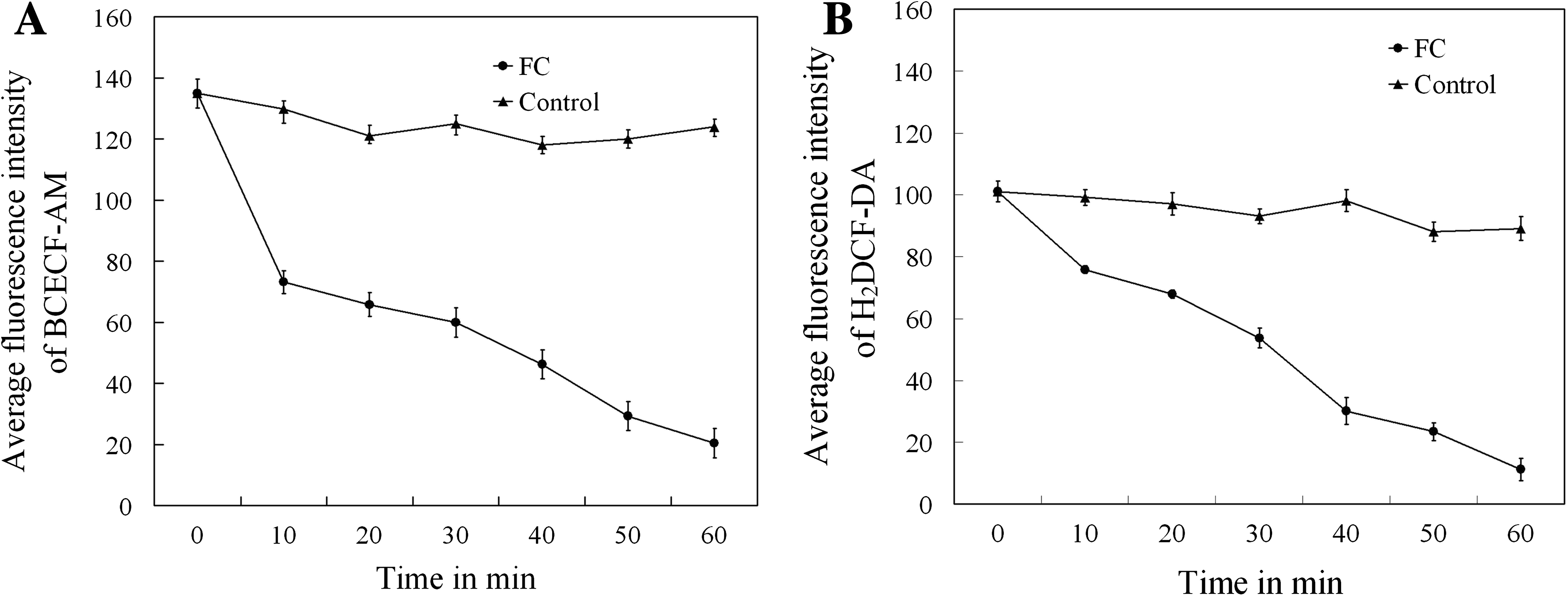

Supplement: Supplementary file 8 — Authors’ original file for figure 8 [file 40529_2012_89_MOESM8_ESM.tif]
